# A Cloud Detection Method for Vertically Pointing Millimeter-Wavelength Cloud Radar

**DOI:** 10.3390/s23218891

**Published:** 2023-11-01

**Authors:** Hai Lin, Jie Wang, Junxiang Ge

**Affiliations:** 1 School of Electronic and Information Engineering, Nanjing University of Information Science & Technology, Nanjing 210044, China; eleclin@nuist.edu.cn (H.L.); wangjie@nuist.edu.cn (J.W.); 2 Institute of Electronics Information Technology and System, Nanjing University of Information Science & Technology, Nanjing 210044, China; 3 Jiangsu Key Laboratory of Meteorological Observation and Information Processing, Nanjing University of Information Science & Technology, Nanjing 210044, China

**Keywords:** MMCR, cloud detection, adaptive filter, noise level, three dimensions

## Abstract

A new method using three dimensions of cloud continuity, including range dimension, Doppler dimension, and time dimension, is proposed to discriminate cloud from noise and detect more weak cloud signals in vertically pointing millimeter-wave cloud radar observations by fully utilizing the spatiotemporal continuum of clouds. A modified noise level estimation method based on the Hildebrand and Sekhon algorithm is used for more accurate noise level estimation, which is critical for weak signals. The detection method consists of three steps. The first two steps are performed at the Doppler power spectrum stage, while the third step is performed at the base data stage. In the first step, a new adaptive spatial filter combined with the Kuwaraha filter and the Gaussian filter, using the ratio of mean to standard deviation as the adaptive parameter, is applied to initially mask the potential cloud signals to improve the detection performance at the boundary of cloud and noise. Simulations of boundary cases were performed to compare our adaptive filter and normal Gaussian filters. Box filters are used in steps two and three to remove the remaining noise. We applied our method to cloud radar observations with TJ-II cloud radar at the Nanjing University of Information Science & Technology. The results showed that our method can detect more weak cloud signals than the usual methods, which are performed only at the Doppler power spectrum stage or the base data stage.

## 1. Introduction

Clouds cover nearly two-thirds of the Earth’s surface and play an important role in climate change, the global radiation budget, and weather forecasting [1,2,3]. The current common remote sensing devices for clouds include weather radar, cloud lidar, and millimeter-wave cloud radar (MMCR) [4,5,6]. These instruments have different application scenarios. With higher spatial and temporal resolution than weather radar and better penetration than lidar, MMCR is the better tool for cloud sensing in both macro and microphysics [7]. Due to the outstanding advantages for cloud research, MMCRs have been deployed on various research platforms, including CloudSat with Cloud Profiling Radar (CPR), the Atmospheric Radiation Program (ARM) with Ka-band Zenith Radar (KAZR), etc. [8,9]. The frequencies of 35 GHz (Ka-band) and 94 GHz (W-band) are the most common operating frequencies of MMCRs, and 94 GHz MMCRs show better detection performance than the 35 GHz (Ka-band) ones because of the shorter wavelength with a stronger cloud particle backscatter [10].

In the early days, starting from the first 94 GHz cloud radar developed by Lhermitter [6], there were two common systems, pulse Doppler (PD) radar with a traveling wave tube amplifier (TWTA) and frequency-modulated continuous wave (FMCW) radar with a solid-state power amplifier (SSPA) with two antennas [6,11,12,13,14,15,16]. Benefiting from recent advances in SSPA technology, pulse Doppler radars with SSPA are increasingly being used in a variety of applications, including the pulse Doppler MMCR. It addresses the shortcomings of the TWTA system, such as size, power requirements, reliability, etc. However, it is still not as sensitive as the TWTA system. Therefore, SSPA pulse Doppler MMCR has to pay extra attention to weak cloud signal detection to improve the overall detection performance compared to the TWTA system.

The simplest method to discriminate cloud from noise is to determine data bins larger than a certain value as cloud signal, which causes a high false-alarm rate (FAR) and missed detection rate (MDR). To decrease both the FAR and MDR, most cloud detection methods utilize the spatiotemporal continuity of the cloud to discriminate the cloud from noise to achieve better FAR and MDR. Some of them perform the detection method at the Doppler power spectrum stage [17,18]. For example, Shupe [17] thought that spectral peaks of cloud signal should have a minimum width (in his use case, seven points) over the noise level. However, for low SNR cases, due to the influence of echo random fluctuations, a random point in the cloud signal spectral peak is easily lower than the noise level, resulting in that segment of the spectrum peak not being recognized as an effective cloud signal peak. Some of them perform the detection at the base data stage (time–height data) [19,20,21,22]. Clothiaux [19,20] developed a classical cloud mask method by analyzing the strength and significance of returned signals. This method consists of two main steps. First, the raw base data which are greater than an estimated mean value of noise plus 1 standard deviation are marked as potentially real. Second, when there are multiple hydrometer bins nearby, the number of bins marked as potentially real should be larger than the one if there is only noise. Therefore, a space–time coherent filter is created to estimate the significance level of the potential hydrometer bin signal to be real. This cloud mask algorithm is operationally used for the ARM MMCR data analysis. Marchand [21] improved this method by marking the raw base data as a different significance level (mean value of noise plus 1, 2, and 3 standard deviations) in step one. The Doppler power spectrum methods utilize the radial continuity of the cloud as a body target and the Doppler velocity broadening properties of the cloud as an aggregate of particles. The base data methods, on the other hand, utilize the cloud as the radial continuity of the body target as well as the temporal continuity (it takes a certain amount of time for a slow-moving body target to pass through the scan area) [20,21]. However, there are few methods that utilize the cloud’s continuity in these three dimensions simultaneously.

In this paper, a new method using three dimensions, including range dimension, Doppler dimension, and time dimension, is proposed to discriminate clouds from noise and detect weak signals in millimeter-wave cloud radar. As a prerequisite for cloud detection, we also develop a new noise level estimation method based on the segment method [23] and Hildebrand and Sekhon (H-S) algorithm [24] to obtain a more stable and accurate noise level estimation. Then, we develop an adaptive filter combined with the Kuwaraha filter and the Gaussian filter, using the ratio of mean to standard deviation as the adaptive parameter to initially mask the cloud signals from noise at the Doppler power spectrum stage. Also, a much lower threshold is used than the one used in common Doppler power spectrum methods. Box filters are used to remove the falsely detected noise at the Doppler power spectrum stage and base data stage, respectively. Observations from the TJ-II cloud radar, a 94 GHz solid-state pulse Doppler MMCR developed by the Nanjing University of Information Science and Technology (NUIST) (Nanjing, China), were used to verify our method.

Our new method has three improvements. The first is the new noise level estimation method. Our noise level estimation method avoids the problem of underprediction caused by the segment method of minimum mean selection and the problem of overprediction caused by the H-S method due to incomplete signal stripping. The computational cost of our noise level estimation method is also more controllable and less than that of the H-S method. The second improvement is the new adaptive filter in the initial cloud signal masking step, which achieves better edge-preserving properties by using the statistical characteristics of the noise and the cloud signal. The third is that the entire detection process has changed from being based on the power spectrum alone or the base data alone to a combination of the two, which utilizes the continuity characteristics of the cloud in a more multi-dimensional way to achieve better detection results.

The TJ-II MMCR is described in Section 2. The noise level estimating method is described in Section 3. The cloud signal detection method is described in Section 4. Simulation and case studies of our method applied to the TJ-II observations are shown in Section 5.

## 2. The TJ-II Radar

**Figure 1:**
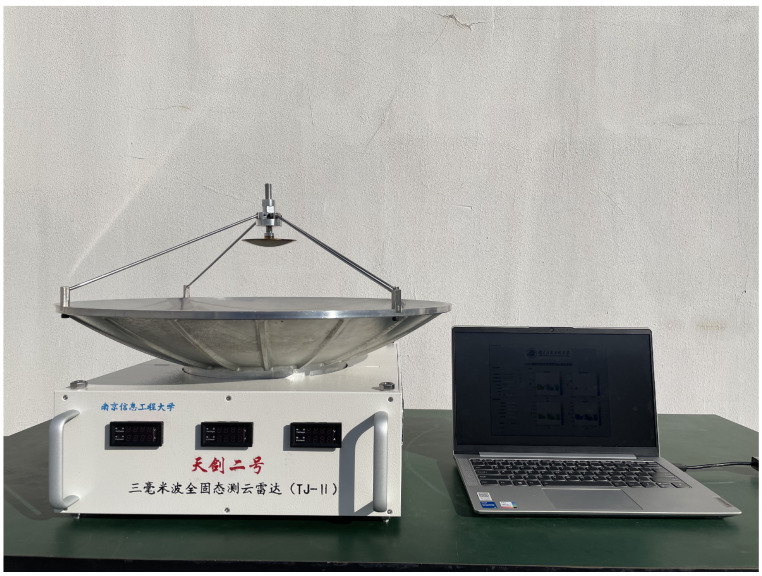
The TJ-II cloud radar.

The NUIST-developed TJ-II millimeter-wave cloud radar is a ground-based vertical sensing cloud radar, as shown in Figure 1. It works at 94 GHz for dual-polarization measurements. It is a pulse Doppler radar using SSPA and has only a single antenna with two polarized ports [25]. Segment pulse detection and pulse compression technology are used to overcome the lower output power of SSPA. Dual PRF technology is used for the velocity dealiasing that comes with the 94 GHz high frequency. The primary purpose of the TJ-II is to provide a small mass and size and low-cost 94 GHz MMCR. It can provide reflectivity, mean Doppler velocity and spectral width, and linear depolarization ratio (LDR). The performance metrics of the TJ-II are listed in Table 1. The peak power of the transmitter is 6 W. The pulse length varies from 1 μs to 20 μs depending on the detection altitude. The detection altitude ranges from 300 m to 15 km, covered by three pulse lengths with a 12 m range gate, providing good support for spatial continuity. The pulse repetition time is between 120 μs and 150 μs to balance the unambiguous range and the Nyquist velocity.

## 3. Noise Level Estimation

**Figure 2:**
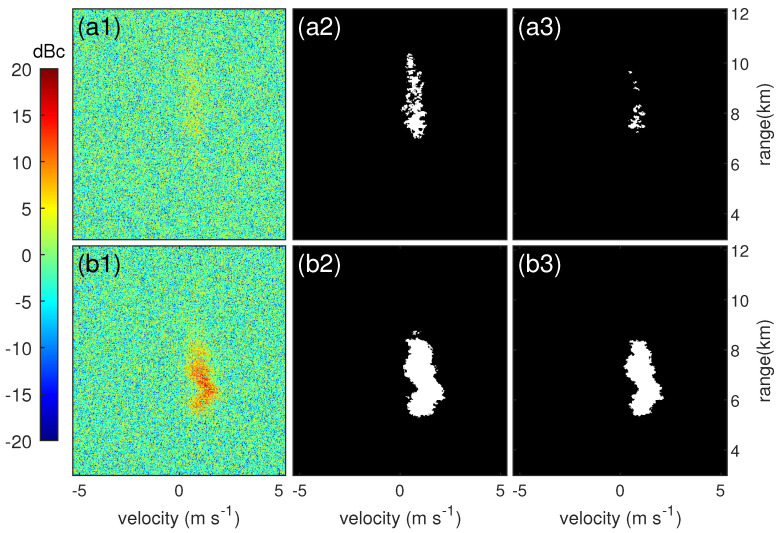
Impact of noise level estimation deviation on weak cloud signals. (**a1**) and (**b1**) are the raw Doppler power spectrum. (**a2**) and (**b2**) are cloud signal mask results with noise levels estimated manually. (**a3**) and (**b3**) are cloud signal mask results with noise levels 1 dB higher than (**a2**) and (**b2**).

Noise level is the average noise power in the Doppler power spectrum, which is essential for discriminating signal from noise. The accuracy of the estimated noise level will directly affect the performance of cloud signal detection, especially for weak cloud signals, and then affect the calculation of the spectral moment. As shown in Figure 2, the deviation of the noise level estimation has a significant impact on the detection result of the weak signal. Even a 1 dB overestimation will cause a large number of weak signals to go undetected. For strong signals, the effect of the deviation is much smaller. Therefore, a robust noise level estimation method with high stability and accuracy is required to enable a more stable detection capability for weak signals.

Starting from the early fixed-value method, a variety of noise level estimation methods have been developed, including the maximum Nyquist velocity method [26], the farthest range gate method [27], the segment method [23], the well-known Hildebrand and Sekhon (H-S) method [24], etc. In the TJ-II use case, the farthest range gate and the maximum Nyquist velocity method do not fit because of the limited detection range and Nyquist velocity. The assumption of no signals at the farthest range gates or maximum Nyquist velocity may fail because of the short detection range and low Nyquist velocity. The segment method can effectively avoid the influence of cloud signals on the noise level estimation, but at the same time, it will increase the fluctuation of the estimated noise level due to the reduction of the number of points used for noise level estimation. What is more, it will also underestimate the noise level due to the selection of the minimum value.

The H-S method seems to be the preferred one, which is based on rigorous theory and widely used in many weather radars. However, there are still some problems. One problem is the uncertainty of the computational complexity. The H-S method is an iterative method and its iterations will vary according to the proportion of the signal in the Doppler power spectrum, making it difficult to accurately estimate its computational load in real-time systems. Its time complexity can vary from O(nk) to $O(n^{2}k)$, where *n* is the number of points in one segment and *k* is the number of segments. This problem can be solved by reserving sufficient computational resources, which may be a bit wasteful. The key problem of the H-S method is the deviation of the estimated noise level for weak signals. The deviation at different SNRs was simulated. The simulation was performed by using a 512-point segment with three-quarter noise bins and one-quarter signal bins. As we know, the cloud signal and noise are both Gaussian signals, while the cloud signal is narrow band and colored and the noise is white. Both their envelopes follow a Rayleigh distribution and their phases follow a uniform distribution. The real part and imaginary part follow a normal distribution, have the same mean and variance, and are uncorrelated. Since a Fourier transformation is a linear operation, the real (*X*) and imaginary (*Y*) spectrum components still follow a normal distribution. The power spectral, *S*, with $S=X^{2}+Y^{2}$, should have a probability density function (PDF), f(S) equal to

(1) $$f\left(S\right)=\lambda e^{-\lambda S}$$

**Figure 3:**
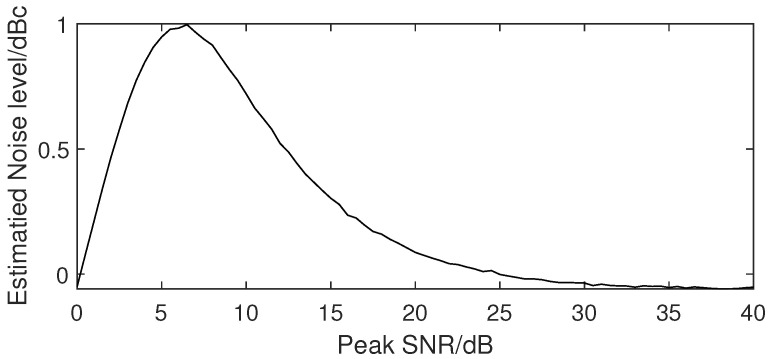
Simulated mean deviation of the estimated noise level by the H-S method with different peak SNRs.

**Figure 4:**
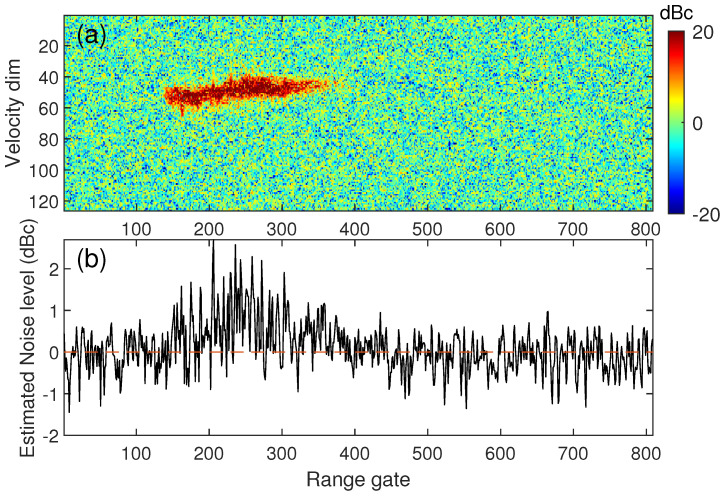
Example of TJ-II observations using the H-S method. (**a**) is the raw Doppler power spectrum, and (**b**) is the noise level estimated by the H-S method.

This PDF is an exponential one, corresponding to a $\chi^{2}$ distribution with two degrees of freedom. Therefore, both noise and cloud signals in the power spectrum used for the simulation should follow an exponential distribution. The generated noise bins follow an exponential distribution with a mean parameter 1 and the generated signal bins also follow the exponential distribution with a mean parameter equal to the SNR value. As shown in Figure 3, the deviations of the estimated noise level with different SNRs were different. The noise level estimated by the H-S method in a low SNR environment was higher than that in a high SNR environment. Around the 6 dB SNR area, the deviations were close to 1 dBc, and it is precisely these areas that are more affected by noise level deviations. The deviation of the estimated noise level with the H-S method was verified by the TJ-II observations, as shown in Figure 4. There was a significant trend of overestimation at the range gate in the presence of the cloud signal. Therefore, the H-S method is not suitable for weak signal cases.

**Figure 5:**
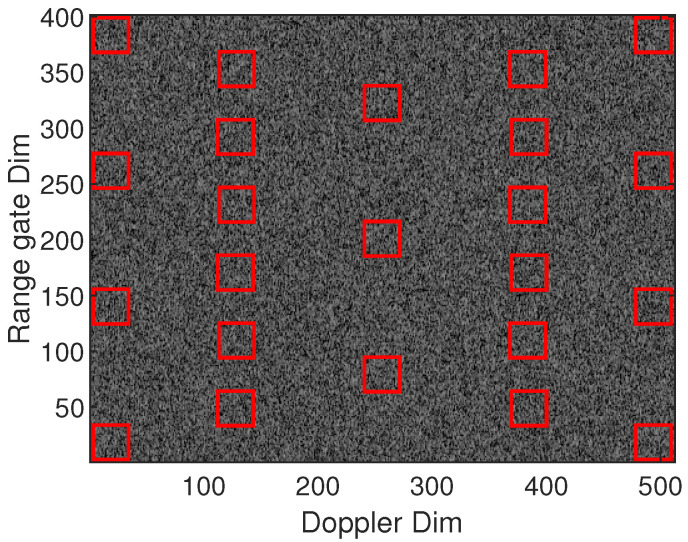
Segment pick example diagram.

To avoid the overestimation by the H-S method, a new method is proposed that combines the segment method and the H-S method to obtain a more stable and accurate noise level estimation. First, several 2-D segments are picked from the Doppler power spectrum in a distributed manner, as shown in Figure 5. Decentralized selection can effectively reduce the likelihood of cloud signals appearing in all segments. Not dividing the entire Doppler power spectrum into 2-D segments is to control the number of segments and then reduce the computational effort. Extending the segment from 1-D to 2-D can effectively increase the number of points for noise level estimation. The stability and accuracy of the estimated noise level are directly related to the number of points used for averaging. According to the central limit theorem (CLT), the standard deviation of the estimated noise level is $\sigma /\sqrt{N}$, where σ is the original standard deviation of the noise and *N* is the number of points used for averaging.

The H-S algorithm is then applied to these segments and the iterations of each segment are limited to five times. The purpose of applying the H-S algorithm here is to distinguish noise segments from mixture segments (or signal segments), not to extract the noise from mixture segments. Therefore, it is not necessary to iterate to the point where the signal bins are completely removed, but only a few iterations are needed to determine whether the segment contains a signal. Limiting the number of iterations also helps to reduce the computational complexity, which becomes O(n), and to keep the overall computational effort manageable.

Finally, select the three segments with the fewest iterations to calculate the noise level. If the iterations are equal, select the one with R2 [24] closest to 1. Selecting multiple segments can further increase the number of points used to estimate the noise level. Therefore, the estimated noise level will be more accurate.

Compared with the native H-S algorithm, our new method uses only the points in the noise segments for noise level estimation instead of the points stripped from mixture segments. It can reduce the computational effort and avoid the overestimation problem of the H-S method in weak mixture segments. It can also avoid the underestimation problem of the segment method which uses the minimum mean value of the segments. In addition, the stability of the estimated noise level will be much higher due to the increased number of points. In the TJ-II application, the number of FFT points is 512 and the size of the two-dimensional window is 31 × 31 with three segments. Therefore the probability of our method predicting noise fluctuations exceeding ±0.5 dBc is $2.65\times 10^{-7}$, while the best one of the H-S method is 0.98%, corresponding to 512 FFT points, according to the CLT.

## 4. Three-Dimensional Cloud-Signal-Mask Method

Except for the simplest single-point threshold determination in the early days, all cloud signal detection methods use the principle of cloud continuity to improve detection performance and reduce FAR and MDR. A common method is to use the continuity of Doppler velocity spectral width of cloud signals [17,18]. Only the spectral segment with $N=2N_{h}+1$ continuous bins all over the threshold $N_{s}$ can be masked as the cloud signal. Typically, the mean cloud thickness is above 1km, and most are above 0.1 km [28,29]. For high range resolution MMCRs, the spatial continuity of the cloud can also be used. Then, two-dimensional detection windows are proposed. The two dimensions mean the range dimension and the Doppler velocity dimension in the Doppler power spectral processing, or the range dimension and the time dimension in the base data stage processing. The two dimensional methods effectively improve the detection performance for weak cloud signals. Since there are three dimensions of continuity for vertically pointing MMCR, in this paper, we propose a cloud signal detection method that uses the three dimensions of continuity to further improve the detection performance for weak cloud signals. Our method is divided into two stages, Doppler power spectrum processing and base data processing.

### 4.1. Doppler Power Spectrum Processing

**Figure 6:**
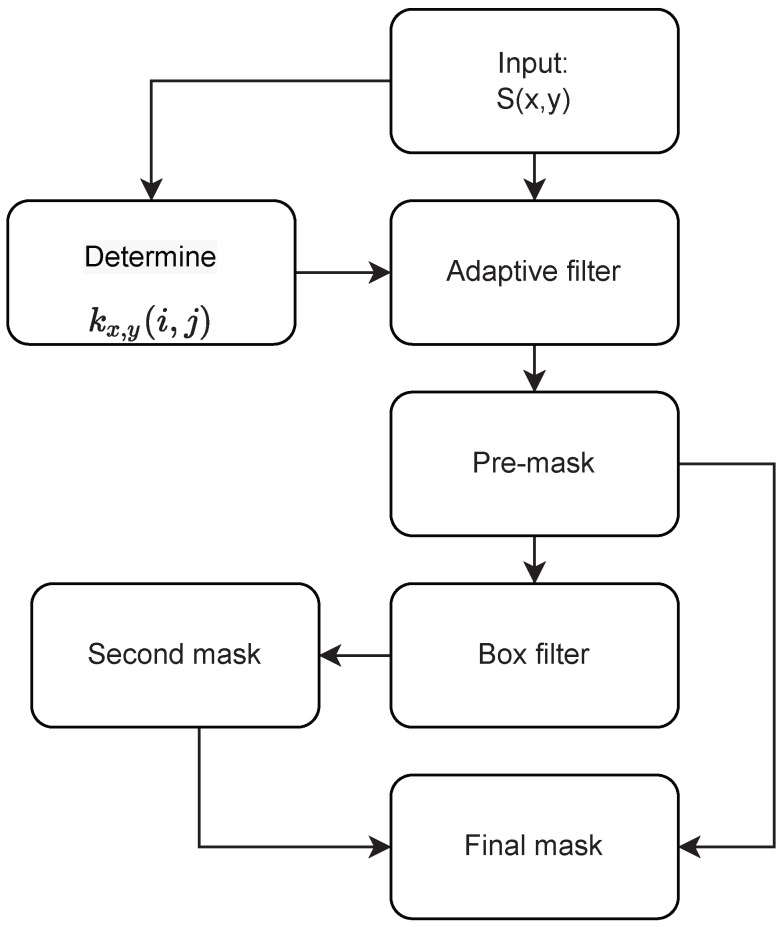
Flow diagram for the Doppler power spectrum stage. S(x,y) is the SNR normalized by noise level, $k_{x,y}\left(i,j\right)$ is the factor matrix of the adaptive filter at point (x,y).

The processing flow of the Doppler power spectrum stage is shown in Figure 6. First, the initial pre-mask processing is performed based on a central pixel weighting scheme, as shown in Equation (2). Here, S(x,y) denotes the SNR value of the spectrum bin at point (x,y), based on the estimated noise level. k(i,j) denotes a normalized filter kernel at point (x,y). $T_{s}$ denotes the threshold and $M_{2w}\left(x,y\right)$ denotes the pre-mask result.

(2) $$M_{2w}\left(x,y\right)=\left\{\begin{array}{ccc} 1, & \sum_{i=-N_{h}}^{N_{h}}\sum_{j=-N_{h}}^{N_{h}}S(x+i,y+j)k(i,j)\geq T_{s} & \\ 0, & else & \end{array}\right.$$

**Figure 7:**
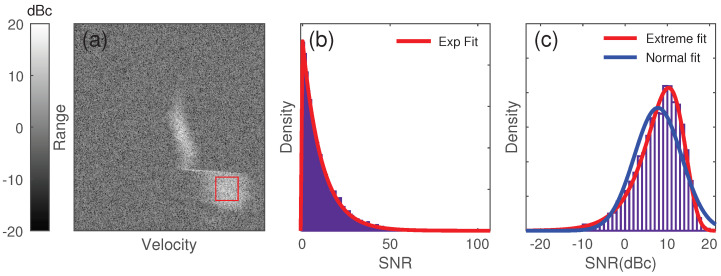
Statistics of manually selected signals within the red rectangle. (**a**) Signals picked from the red rectangle area. (**b**) Linear normalized fit result (**c**) Logarithmic normalized fit result.

An adaptive spatial filter kernel is proposed to improve the performance at the boundary of cloud signal and noise. The proposed adaptive filter combines the Gaussian kernel and the Kuwahara filter and is based on the statistics of the cloud signal and noise. In the TJ-II observations, it was verified that the raw cloud signals in the Doppler power spectrum within a narrow band do follow an exponential distribution, as shown in Figure 7.

The exponential distribution has the property that the ratio of its mean to its standard deviation is 1, while a mixture of signal and noise has the following statistical properties, as shown in Equations (3) and (4). Here, α denotes the proportion of the noise in the mixture. $\mu_{c}$ denotes the mean of the cloud signals. The noise is normalized to 1. Because 0≤α≤1, the ratio is always not greater than 1.

(3) $$\begin{array}{cc} \; & \mu =\alpha +\left(1-\alpha \right)\mu_{c}\\ \; & \sigma^{2}=\alpha +\alpha \left(1-\alpha \right){\left(1-\mu_{c}\right)}^{2}+\left(1-\alpha \right){\mu_{c}}^{2} \end{array}$$

(4) $$\frac{\mu^{2}}{\sigma^{2}}=\frac{1}{1+\frac{2\alpha \left(1-\alpha \right){\left(1-\mu_{c}\right)}^{2}}{{\left[\alpha +\left(1-\alpha \right)\mu_{c}\right]}^{2}}}$$

**Figure 8:**
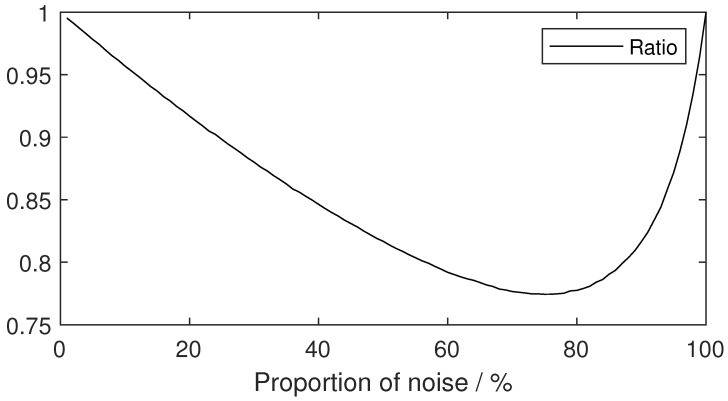
Ratio of the mean and the standard deviation of mixed signal.

Thus, the ratio of the mean to the standard deviation of an area can be used to determine the components, the pure area (noise or signal) or the mixed area (boundary area). As shown in Figure 8, the ratio of the mean to the standard deviation of the mixture is less than 1. The higher the degree of mixing, the lower the ratio, and 0 and 100% represent pure signal and pure noise, respectively. The mean parameters of the exponential distribution were 1 and 3 for noise and signal, respectively.

**Figure 9:**
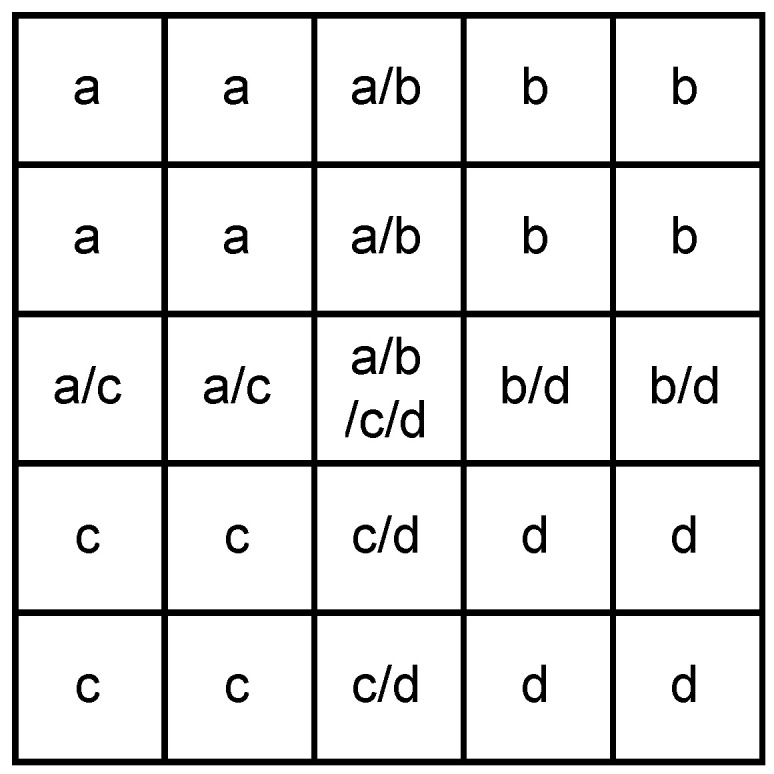
Diagram of subregion division.

The ratio of the mean to the standard deviation is then used as the adaptive parameter. Here is the description of our new filter:

1. Divide the window (2k+1) into four subregions (k+1) as the Kuwahara filter does, as shown in Figure 9
2. Calculate the ratio of the mean $\mu_{i}$ to the standard deviation $\sigma_{i}$ of the four subregions.
  (5) $$r_{i}=\mu_{i}/\sigma_{i}$$
3. Generate four Gaussian filters with a size of 2k+1 and the standard deviations $\sigma_{gi}$ according to the ratio $r_{i}$, and then normalize them with their center pixel.
  (6) $$\sigma_{gi}={r_{i}}^{2}\sigma_{0}$$
4. Fill the four subregions of the final filter with the corresponding subregions of the corresponding Gaussian filter factors. The overlapping regions use the average of the factors.
5. Normalize the final filter and apply it to the original window.

**Figure 10:**
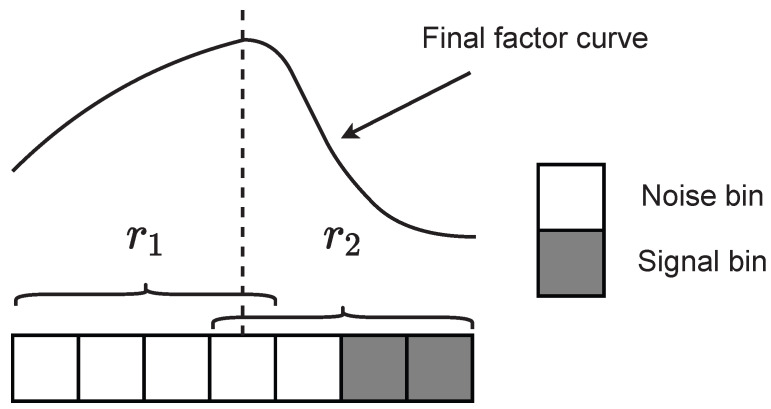
Example of the new filter with 1-D window.

The ratio $r_{i}$ is used to determine the mixing degree of the subregion. The lower the mixing degree ($r_{i}$ closer to 1), the higher the weighting ratio (larger $\sigma_{gi}$). The square in Equation (6) is to enhance the trend. Figure 10 shows a simple example of the filter with a 1-D window. Subregion 1 is all noise, while subregion 2 is a mixture of noise and signal, so $r_{1}$ will be closer to 1 than $r_{2}$. The corresponding Gaussian curves for subregion 1 will be flatter and subregion 2 will be sharper. The total weight of subregion 1 is greater than that of subregion 2. Within subregion 2, the Gaussian curve is sharper, more weighted for noise bins near the center, and less weighted for signal bins at the side.

**Figure 11:**
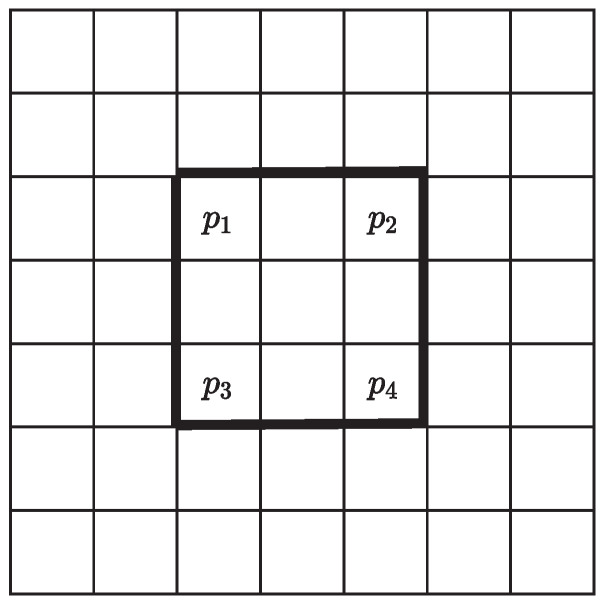
Diagram of shared relationships for a certain subregion.

Due to the additional steps in generating filter coefficients $k_{x,y}\left(i,j\right)$, compared to the filters like the Gaussian and box filters, whose factors are fixed, the computational consumption will increase. In the implementation of the algorithm, certain optimizations can be made to reduce computational complexity. For example, as shown in Figure 11, the 3 × 3 subregion marked by bold will be used in the generation of 5 × 5 adaptive filter related to four bins $p_{1}$ to $p_{4}$. Through optimization, we can only calculate $\mu_{i}$, $\sigma_{i}$, and $r_{i}$ of this subregion once to reduce the computations. Also, when it comes to generating factors of the different Gaussian filters based on $\sigma_{gi}$, the look-up table (LUT) method can be used by saving some pre-generated Gaussian coefficients with different standard deviations and then quantifying $\sigma_{gi}$ to these existing standard deviations.

**Figure 12:**
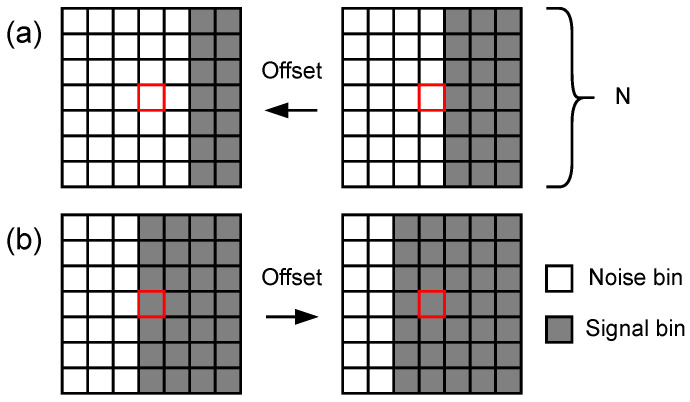
Diagram of half-boundary cases. (**a**) Noise case for FAR evaluation. (**b**) Signal case for MDR evaluation.

Simulation of half-boundary cases was used to evaluate the performance of the new filter, as shown in Figure 12. The red rectangle denotes the central bin. The offset denotes the deviation from the boundary. The case for the FAR evaluation is shown in Figure 12a and offset 0 means that the central bin is the noise bin and falls just at the boundary. The offset direction is toward the noise area. The case for the MDR evaluation is shown in Figure 12b, and offset 0 means that the central bin is the signal bin and falls just at the boundary. The offset direction is toward the signal area.

**Figure 13:**
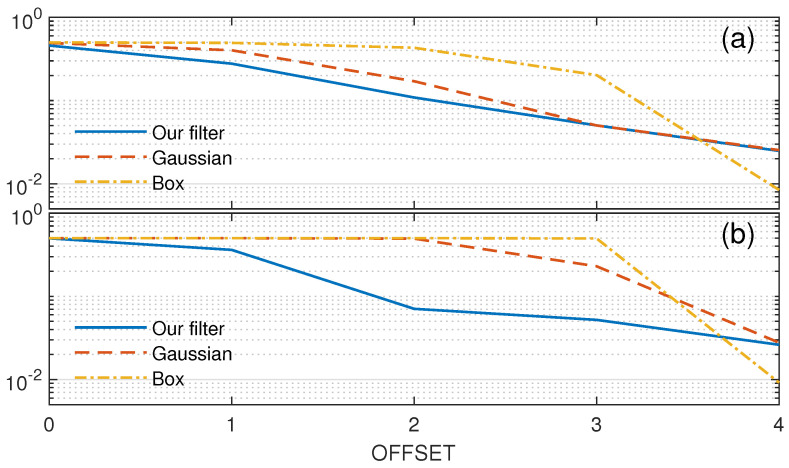
The mean of FAR and MDR with different offsets. (**a**) SNR = 3 (**b**) SNR = 10.

In the simulation, the noise followed an exponential distribution with mean parameter 1. The signal followed an exponential distribution with mean parameters 3 and 10 as weak boundary and strong boundary, respectively. The performance of our filter compared to the standard Gaussian and box filters is shown in Figure 13. The mean error rate, which is the mean of FAR and MDR under the same offset, is used to characterize the overall performance. The window size used in the simulation was 9 × 9, corresponding to the goal that the minimum cloud thickness we want to detect should be over 100 m and then occupy at least 8.3 range gates with 12 m per range gate in TJ-II while 9 is the nearest odd number. The initial standard deviation $\sigma_{0}$ was used in the Gaussian filter, and our filter was 2, corresponding to a common relationship related to the window size, σ=(N−1)/4. The threshold $T_{s}$ was set to 1.25, corresponding to the noise CDF of 0.95 after weighting using the Gaussian filter.

The box filter had the best performance in the signal and noise area but the worst at the boundary. For the low SNR case, such as the cloud top, our filter performed slightly better than the Gaussian filter at the boundary. For the high SNR case, such as the cloud bottom, our filter showed a significant improvement over the Gaussian filter at the boundary. For the signal or noise areas, at offset 4, our filter performed the same as the Gaussian filter.

**Figure 14:**
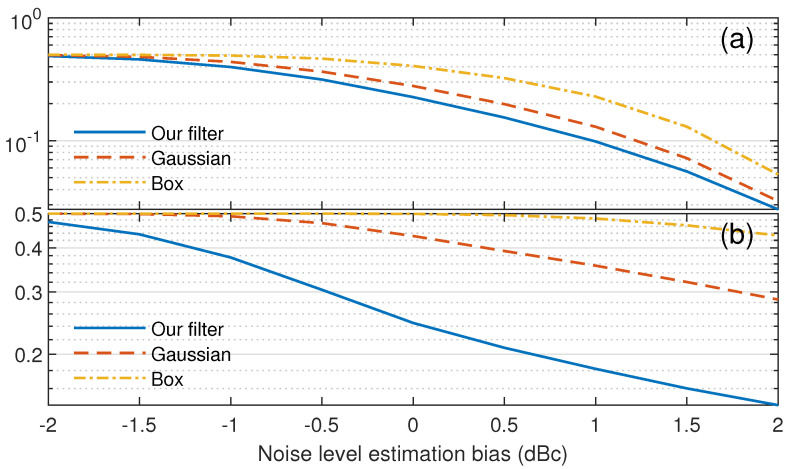
The mean error rate in the boundary region with different noise level estimation biases. (**a**) SNR = 3 (**b**) SNR = 10.

In the simulation above, there is no noise level estimation bias. However, in practice, there is always a noise-level-estimation bias. To show the robustness of our adaptive filter, we also simulated the impact of noise level estimation bias, as shown in Figure 14. To be more concise, further, we use the mean error rate in the boundary region (offset 0 to 3) to illustrate the performance of our adaptive filter on edge preserving. Even though there is a bias in the noise-level estimation, our adaptive filter still has a better edge-preserving performance for both low SNR and high SNR cases. For cases with overestimation of the noise level, which means positive bias in Figure 14, the edge-preserving improvement of our adaptive filter compared to the Gaussian and box filters is increasing. For underestimation cases, the edge-preserving improvement of our adaptive filter weakens but still remains better than the Gaussian and box filters.

After the initial pre-mask processing, most signal blocks are properly masked. There are still some falsely detected noise bins in the noise area. To further reduce the FAR, another mask processing is performed based on the pre-mask result $M_{2w}$ with a box filter, as shown in Equations (7) and (8). Equation (7) masks the potential noise bins as 0 by the threshold $T_{n}$. By performing an AND operation on $M_{2i}$ and $M_{2n}$, the extra masked noise bins in this step, masked as 1 in $M_{2n}$, will not increase the detected signal bins in $M_{2i}$. The window size used in this step should be larger than the one used in the initial mask processing to better remove the falsely detected noise blocks.

Several iterations are then performed to achieve better noise reduction with the box filter. Considering the short-time processing requirements at the Doppler power spectrum stage, only a small number of iterations can be performed. In our case, the window size is set to 15 × 15 and the threshold $T_{n}$ is set to ${\left(N_{h}+1\right)}^{2}$ considering the case of rectangular boundaries and also considering that the minimum cloud thickness we want to detect should occupy 8.3 range gates while 8 is its downward-rounded value, i.e., $N_{h}+1=8$. The iterations are five times. There is always a trade-off in both the window size and threshold selection. These parameters need to be fine-tuned based on demands and long-term observations.

(7) $$M_{2n}\left(x,y\right)=\left\{\begin{array}{ccc} 1, & \sum_{i=-N_{h}}^{N_{h}}\sum_{j=-N_{h}}^{N_{h}}M_{2w}\left(x+i,y+j\right)\geq T_{n} & \\ 0, & else & \end{array}\right.$$

(8) $$M_{f}\left(x,y\right)=M_{2w}\left(x,y\right)\wedge M_{2n}\left(x,y\right)$$

### 4.2. Base Data Processing

After the Doppler power spectrum processing, the signal bins and most of the noise bins are correctly masked, but given the large number of Doppler power spectrum bins, even if the FAR is only 0.0001, there is still a small amount of falsely detected noise bins. Therefore, we perform the third step. Taking advantage of the temporal continuity of the cloud sweeping over the detection region, we use another spatial box filter in the base data stage. Before the box filter is applied, extra quality control (QC) processing is performed. The sum of the number of bins masked in the Doppler power spectrum processing stage is calculated for each range gate. Then, the range gate bins whose sum is larger than the threshold $T_{b}$ are masked as 1 and the others as 0. The QC process can be represented by Equations (9) and (10).

(9) $$M_{b}\left(t,x\right)=\sum_{i=1}^{N_{f}}M_{t,f}\left(x,i\right)$$

(10) $$M_{qc}\left(t,x\right)=\left\{\begin{array}{ccc} 1, & M_{b}\left(t,x\right)\geq T_{b} & \\ 0, & else & \end{array}\right.$$

Here, $M_{t,f}$ denotes the mask result $M_{f}$ of the Doppler power spectral processing at the time *t*. *i* denotes the bin number of the Doppler velocity dim, and $N_{f}$ is the number of FFT points. *t* denotes the time dim or the frame number. *x* denotes the range gate number.

The box filter processing in the base data stage is the same as the one in the Doppler power spectrum stage. The window size depends on the interval time per frame and the range resolution. The shorter the interval is, the larger the size can be used in the time dimension. For the range dimension, we use the same size as in the initial mask processing of the Doppler power spectrum. What is more, since there is more time in the base data stage, the number of iterations can be increased to achieve better noise suppression performance. In our use case, $T_{b}$ is 8, considering that the smallest signal block should have at least 8 bins in the Doppler dimension related to $N_{h}+1=8$ of the previous box filter size 15 × 15 in the Doppler-power-spectrum processing stage. The window size is 9 × 9, which is the same reason as the one of the initial pre-mask processing. The iterations are 15 times.

## 5. Results

### 5.1. Simulation

To test the performance of our cloud detection method, we generated 150 groups of Doppler power spectra. Groups from 20 to 80 contained signal and noise. The size of the Doppler power spectrum is 512 × 280, where 512 is for the Doppler dimension and 280 is for the range dimension. The noise was randomly given by an exponential distribution with mean parameter 1. The signal blocks were also given by exponential distribution with mean parameters 100 (20 dBc), 10 (10 dBc), and 3 (5 dBc), respectively, and the size of the signal blocks was 40 × 40. Another signal block with the size 9 × 9 and the mean parameter 3 was also generated to evaluate the performance for both weak and few signals.

**Figure 15:**
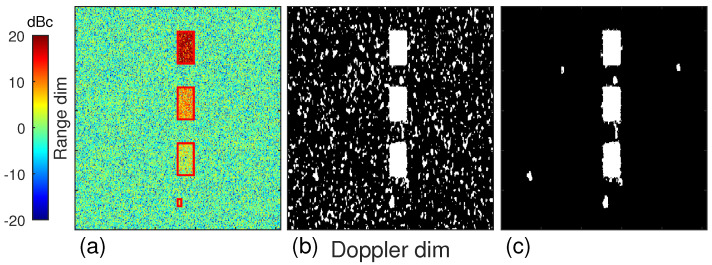
Results of cloud signal mask processing simulation with different block sizes at the Doppler power spectrum stage. (**a**) Raw Doppler power spectrum, (**b**) mask result of step one, (**c**) mask result of step two.

**Figure 16:**
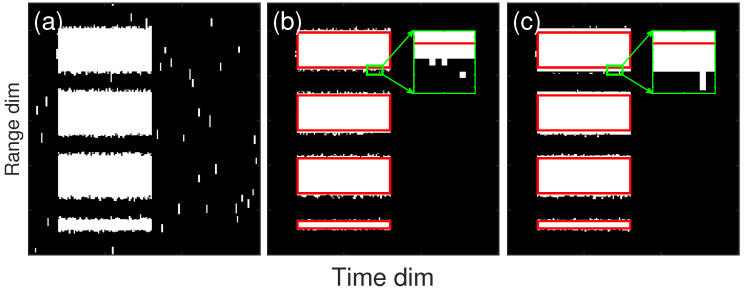
Results of cloud signal mask processing simulation with different block sizes at the base data stage. (**a**) Raw mask result of after Doppler power spectrum stage, (**b**) mask result using our adaptive filter, (**c**) mask result using Gaussian filter.

Figure 15 shows the results of the two steps in the Doppler power spectrum stage. The red rectangles indicate the signal blocks. As shown in Figure 15b, all the signal blocks, even the 9 × 9 one, were well masked, but at the same time, there was a large amount of falsely detected noise bins as well. The box filter in step two removed most of the noise bins, but there was still a small amount of noise that could not be effectively removed due to the formation of noise blocks which are almost bigger than the filter window, as shown in Figure 15c. These noise blocks were removed at the base data stage, using the time dimension, as shown in Figure 16b. In addition, we also simultaneously simulated the results of using a standard Gaussian filter in step one, as shown in Figure 16c, to compare the advantages of our proposed adaptive filter in edge sharpening. As shown in the zoomed part, the falsely detected bins using our adaptive filter at the boundary were significantly fewer than those using the Gaussian filter. The average numbers of falsely detected bins at the boundary were 2.5, 2.5, 2.4, and 0.9 for our filter, and 4, 3.8, 2.9, and 1.3 for the Gaussian filter, corresponding to the SNR 100, 10, 3, and 3 (9 × 9 window size), respectively. The statistics demonstrated the advantage of our adaptive filters over Gaussian filters, especially under strong boundary conditions.

### 5.2. Application to the TJ-II Observations

Our three-dimensional cloud detection method was then applied to the TJ-II observation data on 17 July 2023, at the Huainan Climate and Environment Observatory (HCEO) of the Institute of Atmospheric Physics (IAP), Chinese Academy of Sciences in Huainan. The TJ-II cloud radar operated near another 94 GHz pulse Doppler TWTA cloud radar of IAP. This TWTA cloud radar is more sensitive than TJ-II because of the high-power TWTA and larger antenna. Therefore, we used its result as a reference to evaluate the performance of our algorithm. Because of the time resolution and the range resolution between the TJ-II cloud radar and the IAP cloud radar, we used interpolation to map the results of the IAP cloud radar onto the mesh of TJ-II results.

**Figure 17:**
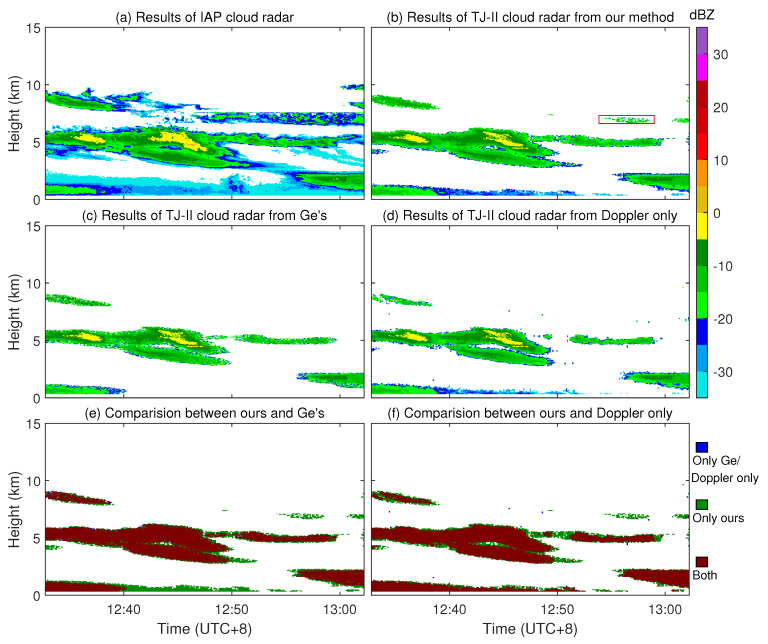
Example of TJ-II observed clouds at the HCEO on 17 July 2023. (**a**) Results of the IAP cloud radar. (**b**) Results of the TJ-II cloud radar using our method. (**c**) Results of the TJ-II cloud radar using Ge’s method. (**d**) Results of the TJ-II cloud radar using the Doppler-power-spectrum-only method. (**e**) Comparision results between our method and Ge’s. (**f**) Comparision results between our method and the Doppler-power-spectrum-only method.

The example of radar reflectivity observed on 17 July 2023, is shown in Figure 17. The detection result of the IAP cloud radar is shown in Figure 17a. The detection result from our method is shown in Figure 17b. The detection result from Ge’s method [22], which is performed only at the base data stage using the raw reflectivity data and is optimized for MMCR, is shown in Figure 17c. The detection result, only performing the same Doppler power spectrum processing as ours with a higher threshold $T_{s}$ 1.55, is shown in Figure 17d. There was still a small amount of falsely detected noise. The comparison of our method with Ge’s method and the Doppler power spectrum only method for the detection results is shown in Figure 17e and Figure 17f, respectively. By comparing Figure 17a and Figure 17b, it can be seen that the sensitivity of TJ-II is indeed lower than that of the IAP cloud radar. Therefore, we can rely on the results of the IAP cloud radar to determine whether certain small detected cloud blocks in TJ-II results are clouds or noise. And, these small blocks are indeed cloud signals, not noise. By comparing Figure 17b, Figure 17c, and Figure 17d, it is easy to see that our method is more powerful than the other two methods, especially for the red rectangular part in Figure 17b, which was not detected by either Ge’s method or the Doppler only method. For Ge’s method, shown in Figure 17c, it also misses the detection of the weak cloud signals between 12:40 and 12:50, adjacent to the blind zone. By further looking at the comparison result shown in Figure 17e and Figure 17f, in the region of the main cloud blocks around 5 km, the results of our method showed a tendency to wrap around the other results, again proving the stronger detection performance of our method.

The statistics based on the results of the IAP cloud radar are shown in Table 2. We use the results of the IAP cloud radar as a reference because it has much higher sensitivity than TJ-II radar. For the results of the TJ-II radar by three different methods, if the bin we detected as the cloud signal was also detected as the cloud signal in the IAP results, we marked it as a true detection. If the bin we detected as the cloud signal was not detected as the cloud signal (be detected as noise) in the IAP results, we marked it as a false detection. If the bin we detected as noise was detected as the cloud signal in the IAP results, we marked it as a missed detection. And, detection rate (DR) is the ratio of the number of bins marked as true detection to the number of bins detected by the IAP cloud radar. FAR is the ratio of the number of bins marked as false detection to the number of bins not detected by the IAP cloud radar. MDR is the ratio of the number of bins marked as missed detection to the number of bins detected by the IAP cloud radar. The low DR values are due to the lower sensitivity of the TJ-II compared with the IAP radar. What is important is the relative value between the three methods. Compared to Ge’s method, our method is able to detect 36.78% more cloud bins. Compared to the Doppler-only method, our method is able to detect 28.44% more cloud bins. For the FAR value, the higher FAR of our method is mainly due to the edge blurring caused by the interpolation process of the IAP results, whose range gate is 30 m and TJ-II’s is 12 m.

## 6. Summary and Dicussion

MMCR has been proven to be an excellent tool for cloud vertical structure sensing. With the development of SSPA, 94 GHz solid-state pulse Doppler MMCR began to appear. It has the advantages of lower cost, size, and stability than the TWTA one, which is favorable for the popularization and application of MMCR. However, it is still limited by the peak transmit power, and its sensitivity still has a certain gap compared with the TWTA one. In order to improve the detection performance, we propose a new cloud detection method based on the observation data of a solid-state pulse Doppler MMCR, TJ-II.

Based on the continuity of cloud in three dimensions, the range dimension (vertical spatial scale), the Doppler dimension (particle swarms), and the time dimension (horizontal spatial scale), our method consists of two phases with three steps: Doppler power spectrum processing and base data processing. In the Doppler power spectrum processing phase, after an improved noise level estimation scheme, we use an adaptive spatial filter for cloud signal detection in the first step. This step focuses on the detection of weak cloud signals and reduces the blurring of the boundary of the cloud signal blocks by using a lower threshold. The remaining two steps remove the falsely detected noise by using a box filter in the Doppler power spectrum stage and the base data stage. The advantage of our adaptive filer in boundary preservation was demonstrated by simulation compared to the Gaussian filter. Compared to the methods that only use two dimensions of continuity, only the range and Doppler dimension, or only the range and time dimension, the advantage of our method that uses three dimensions of continuity in weak cloud detection is verified by the TJ-II observation data.

With long-term observations in the future, the parameters used in our method have room for optimization. Along with the development of TJ-II, some extra quality-control methods are also being studied for better overall performance.

## Figures and Tables

**Table 1:** Performance metrics of the TJ-II MMCR.

Frequency	94 GHz
Transmitter type	Solid-state power amplifier
Peak transmitter power	6 W
Antenna type	Single Cassegrain antenna
Antenna gain	50.8 dBi
Antenna beamwidth	0.45°
Vertical sampling	12 m
Pulse width	1.5/5/20 μs
Pulse repetition time	120/150 μs
FFT points	128/512
Sensitivity	−20 dBZ at 8 km

**Table 2:** Statistics for three methods based on the example.

Method	DR	MDR	FAR
Our method	44.59%	55.41%	0.14%
Ge’s method	32.60%	67.40%	0.02%
Doppler only	34.72%	65.28%	0.04%

## Data Availability

Data related to this article are available upon request to the corresponding author.

## Abbreviations

The following abbreviations are used in this manuscript:

MMCR	Millimeter-wave cloud radar
CPR	Cloud-profiling radar
ARM	Atmospheric Radiation Program
KAZR	Ka-band Zenith Radar
PD	Pulse Doppler
TWTA	Traveling wave-tube amplifier
SSPA	Solid-state power amplifier
FMCW	Frequency-modulated continuous wave
DR	Detection rate
FAR	False-alarm rate
MDR	Missed detection rate
H-S	Hildebrand and Sekhon
NUIST	Nanjing University of Information Science & Technology
PRF	Pulse repetition frequency
LDR	Linear depolarization ratio
SNR	Signal-to-noise ratio
CLT	Central limit theorem
FFT	Fast Fourier transform
CDF	Cumulative distribution function
PDF	Probability density function
QC	Quality-control
HCEO	Huainan Climate and Environment Observatory
IAP	Institute of Atmospheric Physics
